# Low and differential polygenic score generalizability among African populations due largely to genetic diversity

**DOI:** 10.1016/j.xhgg.2023.100184

**Published:** 2023-02-13

**Authors:** Lerato Majara, Allan Kalungi, Nastassja Koen, Kristin Tsuo, Ying Wang, Rahul Gupta, Lethukuthula L. Nkambule, Heather Zar, Dan J. Stein, Eugene Kinyanda, Elizabeth G. Atkinson, Alicia R. Martin

**Affiliations:** 1 Global Initiative for Neuropsychiatric Genetics Education in Research (GINGER), Harvard T.H. Chan School of Public Health, Department of Epidemiology, Boston, MA, USA; 2 MRC Human Genetics Research Unit, Division of Human Genetics, Institute of Infectious Disease and Molecular Medicine, Faculty of Health Sciences, University of Cape Town, Observatory 7925, South Africa; 3 Department of Psychiatry, College of Health Sciences, Makerere University, Kampala, Uganda; 4 Department of Psychiatry, Faculty of Medicine and Health Sciences, Stellenbosch University, Cape Town, South Africa; 5 Mental Health Project, Medical Research Council/Uganda Virus Research Institute (MRC/UVRI) & London School of Hygiene and Tropical Medicine (LSHTM), Uganda Research Unit, Entebbe, Uganda; 6 Department of Psychiatry and Neuroscience Institute, University of Cape Town, Cape Town, South Africa; 7 South African Medical Research Council (SAMRC) Unit on Risk and Resilience in Mental Disorders, Cape Town, South Africa; 8 Analytic and Translational Genetics Unit, Massachusetts General Hospital, Boston, MA 02114, USA; 9 Stanley Center for Psychiatric Research, Broad Institute of MIT and Harvard, Cambridge, MA 02142, USA; 10 Program in Medical and Population Genetics, Broad Institute of MIT and Harvard, Cambridge, MA 02142, USA; 11 Program in Biological and Biomedical Sciences, Harvard Medical School, Boston, MA 02115, USA; 12 Department of Paediatrics and Child Health, Red Cross Children’s Hospital and Medical Research Council Unit on Child and Adolescent Health, University of Cape Town, Cape Town, South Africa; 13 Department of Molecular and Human Genetics, Baylor College of Medicine, Houston, TX 77030, USA

**Keywords:** polygenic scores, Africa, GWAS, health disparities, global health, population genetics

## Abstract

African populations are vastly underrepresented in genetic studies but have the most genetic variation and face wide-ranging environmental exposures globally. Because systematic evaluations of genetic prediction had not yet been conducted in ancestries that span African diversity, we calculated polygenic risk scores (PRSs) in simulations across Africa and in empirical data from South Africa, Uganda, and the United Kingdom to better understand the generalizability of genetic studies. PRS accuracy improves with ancestry-matched discovery cohorts more than from ancestry-mismatched studies. Within ancestrally and ethnically diverse South African individuals, we find that PRS accuracy is low for all traits but varies across groups. Differences in African ancestries contribute more to variability in PRS accuracy than other large cohort differences considered between individuals in the United Kingdom versus Uganda. We computed PRS in African ancestry populations using existing European-only versus ancestrally diverse genetic studies; the increased diversity produced the largest accuracy gains for hemoglobin concentration and white blood cell count, reflecting large-effect ancestry-enriched variants in genes known to influence sickle cell anemia and the allergic response, respectively. Differences in PRS accuracy across African ancestries originating from diverse regions are as large as across out-of-Africa continental ancestries, requiring commensurate nuance.

## Teaser

Majara et al. show that polygenic scores generalize variably across diverse populations due to genetic differences between target and discovery cohorts. Multi-ancestry discovery GWAS typically improve prediction accuracy in underrepresented populations more than an increase in the European sample size, but not necessarily when sample sizes are imbalanced.

## Introduction

Genome-wide association studies (GWASs) have yielded important biological insights into the heritable basis of many complex traits and diseases.^1^ However, the vast majority of studies have been conducted in populations of European descent, potentially limiting generalizability across diverse populations.^2^^,^^3^^,^^4^^,^^5^^,^^6^ Genome-wide significant SNP associations with phenotypes spanning a wide range of genetic architectures have consistently replicated across populations in both direction and effect size, with few examples of heterogeneous effect sizes.^7^^,^^8^^,^^9^ However, previous studies that have compared the association between genetically predicted versus measured phenotypes in diverse populations using polygenic risk scores (PRSs) have found that PRS accuracy decreases with increasing genetic distance between the GWAS discovery and PRS target cohorts.^4^^,^^10^^,^^11^ This seeming paradox highlights that while variant-level associations consistently replicate across populations, genome-wide aggregate measures are more predictive but less generalizable.^12^ Since the earliest applications of PRS in human genetics, these concepts—coupled with Eurocentric study biases—have resulted in PRSs that are most accurate in European ancestry populations and least accurate in African ancestry populations.^13^ These study biases and phenomena continue to replicate a decade later, with several-fold differences in prediction accuracy of many traits between European and non-European ancestry populations.^4^

Quantifying PRS generalizability within and among African populations requires considerable nuance, as they represent the most genetically diverse populations globally, with more than a million more genetic variants per person than out-of-Africa populations.^14^ Populations collected even within the same geographic regions of Africa have complex demographic histories with complicated patterns of admixture and population structure.^15^^,^^16^^,^^17^^,^^18^ Further, African ancestry populations experience vastly different environments within versus outside continental Africa as well as more locally among diverse communities, countries, and regions of Africa. These differences provide unique epidemiological opportunities to query the impacts of vastly differing environments on PRS accuracy. Previous empirical analyses and theoretical work fundamentally informs how demographic history and environmental variation interplay to produce PRS heterogeneity in traditionally underserved populations.^19^^,^^20^^,^^21^^,^^22^^,^^23^

The inclusion of African ancestry participants in large-scale genetic studies is uniquely important for many reasons. They have the lowest life expectancies globally,^24^^,^^25^ receive the lowest access to and quality of medical care in the United States,^26^ and are the most underserved by genetic technologies.^6^^,^^27^ A more nuanced understanding of PRS transferability will critically inform which populations are currently the most underserved and thus where building genetic studies and resources will have the biggest benefits globally.

There are also clear benefits to including African populations in statistical genetics efforts. Because humans originated in Africa, populations from Africa have the most genetic diversity among global populations,^14^^,^^28^^,^^29^ such that more genotype-phenotype associations are expected in Africa than can be found elsewhere. African American individuals have been shown to contribute disproportionately to GWAS findings,^2^ making up 2.8% of GWAS participants but contributing 7% of trait associations. African ancestry populations also have shorter blocks of linkage disequilibrium, which improves resolution to fine-map causal variants.^30^ PRS accuracy is lowest in African ancestry populations due to GWAS study biases,^4^ but when GWASs include these and other diverse populations, PRS predict traits such as schizophrenia more accurately across all populations compared with single-ancestry GWASs.^31^

In this study, we have investigated how PRSs generalize within and among diverse African populations in simulations and with empirical genotype-phenotype data for dozens of quantitative traits. We first simulated genetic effects and computed genetic risk prediction accuracy using data from two African datasets: the African Genome Variation Project (AGVP) and the Africa Wits-INDEPTH partnership for Genomic Studies (AWI-Gen) project. We then calculated PRS using publicly available GWAS summary statistics from predominantly European ancestry populations to (1) quantify PRS accuracy for five physical and psychosocial traits among populations in the Drakenstein Child Health Study (DCHS) of South Africa, a birth cohort study; and (2) compare PRS accuracy for 34 quantitative traits across the Ugandan General Population Cohort (GPC) versus ancestrally diverse UK Biobank participants. Our results highlight the disproportionate benefits of genetic studies in diverse African populations to improve trait prediction. Further, while PRSs hold promise as biomarkers in precision medicine, a critical prerequisite is equitable accuracy in diverse populations to avoid exacerbating existing health disparities.

## Materials and methods

### Genetic and phenotypic data

Total counts of individuals by population and/or study are shown in Table S2.

#### 1000 Genomes Project

1000 Genomes Project data from the phase 3 integrated call set were accessed and used as a reference panel and for phasing and imputation.^14^

#### Human Genome Diversity Project

Genotype data for samples from the Human Genome Diversity Project (HGDP) was publicly available on the Illumina HumanHap650K GWAS array on hg18.^32^ We lifted over the genotype data to the hg19 genome build using hail (http://hail.is).

#### African Genome Variation Project

As described previously,^33^ the AGVP data consist of dense genotype data from 1,481 individuals from 18 ethnolinguistic groups from Eastern, Western, and Southern Africa when including the Luhya and Yoruba from the 1000 Genomes Project.^14^ When accessed from the European Genome-Phenome Archive (EGA:EGAD00010001047), “Ethiopian” is the provided population label encompassing the Oromo, Amhara, and Somali groups. After collapsing these groups and counting the 1000 Genomes data separately, 1,307 individuals from 14 populations are uniquely represented in the AGVP, and 2,504 individuals from 26 populations are represented in the 1000 Genomes Project data (661 individuals from seven populations are in the AFR super population grouping).

#### Africa Wits-INDEPTH partnership for genomic studies

AWI-Gen is a study that investigates the relationship between genetics and the environment in causing cardiometabolic disease in sub-Saharan Africa with study participants from Burkina Faso, Ghana, Kenya, and South Africa.^34^^,^^35^ It is a partnership between the University of Witwatersrand (Wits) in Johannesburg, South and the International Network for the Demographic Evaluation of Populations and Their Health (INDEPTH). Ethical approval was obtained from the Human Research Ethics Committee of the University of the Witwatersrand (Protocol Number: M121029 and from the institutions of the respective centres that are in the international network.

#### Drakenstein Child Health Study in South Africa

The DCHS is an ongoing, multidisciplinary population-based birth cohort study in the Drakenstein area in Paarl (outside Cape Town, South Africa), that obtained ethical approval from the Faculty of Health Sciences Research Ethics Committee at the University of Cape Town (401/2009) and the Western Cape Provincial Research committee.^36^^,^^37^^,^^38^ After providing informed consent, pregnant women were enrolled during their second trimester (20–28 weeks’ gestation); maternal-child dyads were then followed through childbirth and longitudinally thereafter. Enrollment occurred from March 2012 to March 2015 at two primary health care clinics: TC Newman (serving a predominantly mixed ancestry population) and Mbekweni (serving a predominantly Black African population). Women were eligible to participate in the DCHS if they attended one of the study clinics, were at least 18 years of age, and intended to remain residing in the study area. Ancestral diversity computed using PCA with genetic data is shown in Figure S5 with corresponding self-reported ethnicity (“Mixed” versus “Black/African”).

#### Uganda General Population Cohort

The rural Uganda GPC of MRC/UVRI & LSHTM Uganda Research Unit was set up in 1989 initially to monitor the HIV epidemic among adults, children, and adolescents, after obtaining ethical approval the Uganda Virus Research Institute Science and Ethics committe and the Ugandan National Council of Science and Technology. It's mandate has since expanded to include other medical conditions.^39^ The “original GPC” is located in the sub-county of Kyamulibwa in rural southwestern Uganda with activities having recently been expanded to the neighboring two peri-urban townships of Lwabenge and Lukaya. The “original GPC” includes about 10,000 adults and about 10,000 children and adolescents. In 2011, genotype data were generated on more than 5,000 adult participants from nine ethnolinguistic groups using the Illumina HumanOmni2.5 BeadChip at the Sanger Wellcome Trust Institute.^39^^,^^40^

#### UK Biobank

The UK Biobank enrolled 500,000 people aged between 40 and 69 years in 2006–2010 from across the country, as described previously.^41^ A more detailed description of the cohort is available on their website: https://www.ukbiobank.ac.uk/. We analyzed phenotypes that overlapped with those studied in the Uganda GPC.

### Ancestry analysis in the UK Biobank

As described previously,^41^ the UK Biobank consists of approximately 500,000 participants of primarily European ancestry who have thousands of measured or reported phenotypes. To assess polygenic score accuracy across diverse ancestries, we identified populations of ancestral groups at two levels: (1) among continental groups, and (2) among regions in Africa. To define continental ancestries, we first combined reference data from the 1000 Genomes Project and HGDP. We combined these reference datasets into continental ancestries according to their corresponding meta-data (Table S5). We then ran PCA on unrelated individuals from the reference dataset. To partition individuals in the UK Biobank based on their continental ancestry, we used the PC loadings from the reference dataset to project UK Biobank individuals into the same PC space. We trained a random forest classifier given continental ancestry meta-data (AFR = African, AMR = admixed American, CSA = Central/South Asian, EAS = East Asian, EUR = European, and MID = Middle Eastern) based on the top six PCs from the reference training data. We applied this random forest to the projected UK Biobank PCA data and assigned initial ancestries if the random forest probability was >50% (similar results obtained for p > 0.9), otherwise individuals were dropped from further analysis.

Next, we further partitioned African ancestry individuals using the same random forest approach as above but without further probability thresholding using African ancestry reference data from AGVP, HGDP, and the 1000 Genomes Project. We partitioned these reference data into UN regional codes with an additional region for Ethiopian populations given their unique population history and collapsing in AGVP data (Admixed, Central, East, Ethiopia, South, and West Africa), as shown in Table S5. PCA with reference data at the continental and subcontinental level within Africa are shown in Figures S10 and S11.

### Phasing and imputation

We used the Ricopili pipeline to conduct pre-imputation quality control (QC) and perform phasing and imputation for AGVP and the Uganda GPC.^42^ This pipeline was also used on the DCHS data, as described previously.^43^ Briefly, we phased the data using Eagle 2.3.5 and imputed variants using minimac3 in chunks ≥3 Mb. The 1000 Genomes phase 3 haplotypes were used as the reference panel for phasing and imputation. For the AGVP, we used strict best guess genotypes where a variant was called if it had a probability of p > 0.8 and a missing rate less than 0.01 and MAF >5%. Then, variants with MAF <0.001 were excluded from the dataset. For Uganda GPC, we used combined best guess genotypes where a variant was called if it had a probability p > 0.8 or set to missing otherwise. Then, SNPs were filtered to keep sites with missingness <0.01 and MAF >0.05. We used genotype dosages when computed PRS.

### PCA

Only SNPs with high imputation quality (INFO >0.8) were considered for principal-component analysis. We computed the first 20 principal components using plink with the --pca flag for autosomal SNPs MAF >0.05 and individual missingness <0.05.

### Simulation setup

We used two independent simulation strategies for two African datasets: AGVP and AWI-Gen. The choice of simulation strategy was informed by the sample size of each dataset. We used the simulation strategy previously used by Scutari et al.^11^ for AGVP, and the infinitesimal model for simulations in AWI-Gen. To test the PRS prediction accuracy within and across African populations, we simulated four quantitative traits while varying heritabilities (*h*^*2*^ = 0.1, 0.2, 0.4 and 0.8) for both AGVP and AWI-Gen as follows.

#### AGVP

We randomly assigned an effect size to 5, 20, 100, 2,000, 10,000, and 50,000 causal variants, respectively. We then calculated an individual’s “true” polygenic risk as the sum of all causal effects using the --score flag in PLINK v1.07B.^44^ True polygenic scores were standardized to a mean of zero and standard deviation of 1. To account for the contribution of environmental risk factors, we assigned environmental effects from a normal random distribution (mean = 0 and SD = 1). The phenotype was generated according to its heritability as the weighted sum of the true polygenic risk and a random environmental effect as below:

$$phenotype=h^{2}\times true\;polygenic\;risk+\left(1-h^{2}\right)\times environmental\;effect$$

**Figure 1:**
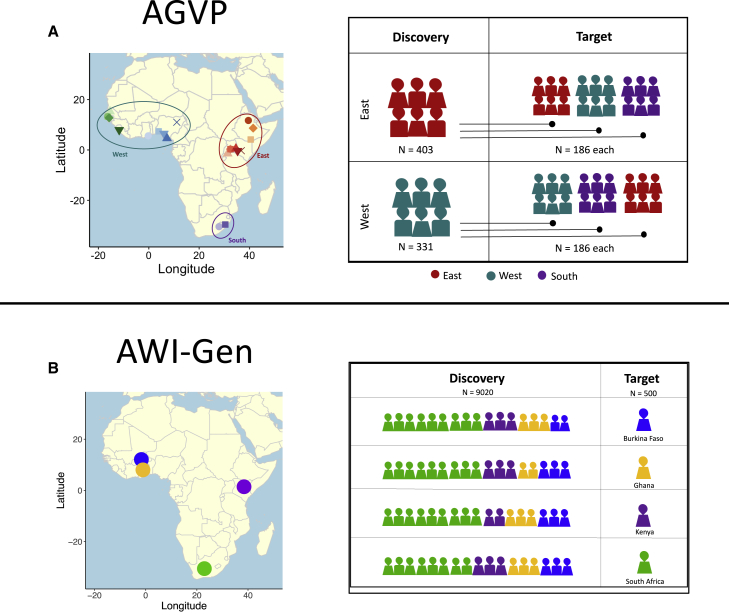
Simulation strategy overview (A) We used AGVP for simulations in West, East, and South African populations that were grouped based on the United Nations geoscheme groupings. Each group was divided into discovery and target subgroups. GWAS discovery cohorts included East (n = 403) and West (n = 331) African individuals, which were independent of each target cohort (n = 186 individuals per region). South African individuals were excluded from the discovery population due to the limited total sample size (two populations and 186 individuals total). (B) We used AWI-Gen for simulations in Burkina Faso (n = 1703), Ghana (n = 1,661), Kenya (n = 1,701), and South Africa (n = 4,455). For these simulations we withheld 500 individuals from each of the groups, which were used as the target cohort. The GWAS discovery cohort included the 9,020 individuals who were not in the target cohort. Each figure represents roughly 500 individuals. BF, Burkina Faso; SA, South Africa.

We then conducted GWAS for the simulated phenotypes by splitting the AGVP dataset into three groups broadly representing the three geographical areas from which samples were obtained: East (n = 589), West (n = 517), and South Africa (n = 186, Figure 1). To allow for the quantification of PRS prediction accuracy across the geographical regions, each group was further split into discovery and target cohorts. The size of the target cohorts was maintained at n = 186 across all groups, while the discovery cohort consisted of all remaining individuals (East n = 403, West n = 331, and no South Africans). We conducted a linear regression for all the simulated traits for the East and West discovery datasets, controlling for the first 20 principal components.

#### AWI-Gen

We assigned genetic effects to variants based on their minor allele frequency. The effects were calculated based on the relationship between effect size and minor allele frequency as shown by Schoech et al.^45^ The “true” individual’s polygenic risk was calculated in the same way as it was for AGVP, so was the environmental risk factor and the phenotypes. To be able to conduct GWAS, we split AWI-Gen into discovery and target sets such that each discovery set had 9,020 samples and each target 500 (Figure 1). For each discovery-target split, we alternately withheld 500 samples from one of the four countries (Burkina Faso, Ghana, Kenya, and South Africa). We then conducted GWAS for each of the discovery datasets.

For each discovery cohort in AGVP and AWI-Gen, we obtained independent SNP sets by clumping SNPs from corresponding summary statistics files with an *r*^2^ value greater than 0.1 using in-sample linkage disequilibrium (LD) and within 500 kb of each other in n PLINK v1.07, were obtained for each discovery cohort. The effect sizes from these SNP sets were used as weights to compute PRSs for all corresponding target datasets for a range of p values (5e-08, 1e-06, 1e-04, 1e-03, 1e-02, 0.05, 0.1, 0.2, 0.5, and all). PRS was calculated as the sum of all SNPs multiplied by their effect sizes.

### Heritability estimation

For the first set of heritability estimation analyses, we relied on heritability estimates of 34 quantitative traits computed previously for the Ugandan GPC dataset.^46^ For the UK Biobank, we computed heritability estimates for the same traits using LD score regression with the default model (i.e., without any functional annotations)^47^ and using population-matched LD score references from European populations downloaded from the authors’ website (https://data.broadinstitute.org/alkesgroup/LDSCORE/). Due to the difference in study design and heritability estimation methods used for UK Biobank and Uganda GPC, we could not directly compare heritability estimates between the two cohorts. For more comparable estimates, we computed heritability estimates across the 34 quantitative traits in both the Ugandan GPC and UK Biobank using a randomized multi-component Haseman-Elston estimator (RHE-mc^48^) over unrelated individuals. This method offers improved power over summary statistic methods (e.g., LD score regression), which show very large standard errors with small sample sizes, with improved computational tractability when operating at biobank scale over GREML-based approaches.

To improve comparability across cohorts, we used parallel QC approaches in both cohorts after restricting to unrelated individuals (N = 2,234 in GPC). Namely, in UK Biobank, we filtered genotypes to those outside the MHC region that were defined with MAF ≥0.01 for which we did not observe significant deviation from Hardy-Weinberg equilibrium (p_hwe ≥ 1e-7) and only used genotypes that passed these criteria across all ancestry groups. For the Ugandan GPC analysis, we applied the same filters as above (i.e., variants outside MHC with MAF ≥0.01 and without significant deviation from Hardy-Weinberg equilibrium). We also restricted the analysis to SNPs with imputation INFO score >0.3 to match the approach taken for the GWAS conducted by Gurdasani et al.,^46^ resulting in 3,627,507 SNPs passing QC.

To account for differences in heritability as a function of LD and MAF, we performed multi-component analyses with a 4 × 2 grid of LD and MAF bins defined in Ugandan GPC and per-population in UK Biobank. LD scores in the Ugandan GPC were computed in LD score regression using all imputed SNPs with MAF >0.005 from unrelated samples, while those in the UK Biobank were computed using SNPs with INFO >0.8, MAC >20 with subsequent covariate correction for age, sex, age∗sex, age^2^, age^2^ ∗sex, and the first 20 genotype PCs in each population. LD score bins in both cohorts were computed as membership in quartiles of the LD score distribution. MAF bins in both cohorts were defined as MAF ≤0.05 and MAF >0.05.

We included standard GWAS covariates as fixed-effects covariates for heritability estimation in both Ugandan GPC and UK Biobank, namely age, sex, age∗sex, age^2^, age^2^ ∗sex, and the first 20 genotype PCs in each cohort and in each population in the UK Biobank. We ran RHE-mc with 50 random vectors and 100 jackknife blocks in the Uganda GPC and among UK Biobank non-EUR populations; and used 10 random vectors with UK Biobank European samples due to high computational complexity.

### Polygenic score calculation

#### Pruning and thresholding

All PRSs were calculated in plink2 or in hail using custom scripts. For pruning and thresholding approaches, all clumping was done in plink2 using an LD threshold of *r*^2^ = 0.1 and a window size of 500 kb with discovery cohort population-specific reference panels. We calculated PRS using plink2 with the --score and --q-score-range flags for AGVP simulations and DCHS. We wrote custom scripts in hail (http://hail.is) to calculate PRS in the Uganda GPC and UK Biobank data due to the larger sample sizes (see web resources). For imputed genotypes, we used SNP dosages in PRS calculations. We computed 10 PRSs for each analysis using the following p-value thresholds: 1, 0.5, 0.2, 0.1, 0.05, 0.01, 1e-3, 1e-4, 1e-6, 5e-8. The PRS that explained the most phenotypic variance is shown in most figures.

We calculated PRS accuracy for continuous traits computed with custom scripts in R (Web resources). For AGVP simulations and DCHS (because all participants were mothers of a similar age), we included the first 10 PCs as covariates when computing the partial *R*^2^ specifically attributable to the PRS. For Uganda GPC data, we included age, sex, and the first 10 PCs when computing partial *R*^2^ of the PRS. For consistency with the GWAS that were run in UK Biobank previously^49^ and here with a holdout target set, we included, age, sex, age^2^, age∗sex, age^2^∗sex, and the first 10 PCs as covariates when computing the PRS partial *R*^2^. (The UK Biobank European GWAS included 20 PCs, but fewer were used here due to the particularly small sample sizes of some other target ancestry groups, Table S5, coupled with minimal population structure observed in PCs lower than PC10.) As described in Table S5, we included up to 351,194 European ancestry participants in a GWAS, withholding up to 9,947 European ancestry participants as a target cohort as well as up to the following numbers of participants with corresponding ancestries: 8,426 African, 1,099 Admixed American, 10,084 Central/South Asian, 2,753 East Asian, and 1,553 Middle Eastern individuals. Other participants in the UK Biobank but not included in these analyses had either second-degree relatives or closer with participants included in analysis or were ancestry outliers.

#### PRS accuracy evaluation (incremental *R*^2^)

To evaluate prediction accuracy, we calculated incremental *R*^2^. Specifically, we compared two models:

H_0_: Phenotype ∼ covariates.

H_1_: Phenotype ∼ PRS + covariates.

The incremental *R*^2^ calculates the change in *R*^2^ between H_1_ and H_0_, indicating the change in model accuracy attributable to the PRS. We used adjusted *R*^2^, which ensures that the model that includes PRS does not outperform the model without PRS simply because more terms were included. All error bars show 95% confidence intervals calculated from bootstrapping. Specifically, for each iteration of 100 bootstrap replicates, we resampled with replacement each individual’s full set of phenotypes, covariates, and polygenic scores, then ran the same models described above. The 95% confidence interval was determined by the 2.5% and 97.5% quantiles.

#### Relative comparisons of PRS across populations

We compared PRS accuracy across populations by computing relative accuracies (RAs) with respect to a baseline European ancestry PRS. For pruning and thresholding PRS, we computed RA as the ratio between the maximum *R*^2^ in the population of interest versus the maximum *R*^2^ in the European baseline comparison (i.e., for the same phenotype). Across traits, we computed median absolute deviation (MAD), i.e., the median of the absolute deviations from the median.

#### PRS-CS versus pruning and thresholding

We compared the prediction accuracy of the pruning and thresholding method to that of PRS-CS, a Bayesian method that has been shown to improve PRS prediction accuracy across diverse populations.^50^ To do this, we applied PRS-CS-auto to generate scores for the same 34 quantitative traits that were evaluated using pruning and thresholding. We maintained the same discovery cohort from the UK Biobank, i.e., 351,194 European ancestry individuals and evaluated prediction accuracy in two target cohorts: (1) continental ancestry groups from the UK Biobank comprising 9,947 European ancestry holdout sample, as well as ∼24,000 non-European ancestry individuals, and (2) the Ugandan GPC. We used European ancestry from the UK Biobank as the reference panel. Relative accuracy was calculated as the ratio between the maximum *R*^2^ for pruning and thresholding or *R*^2^ for PRS-CS versus the maximum *R*^2^ in the European population for each trait.

#### Observed versus predicted PRS accuracy

To evaluate the efficacy of PRS accuracy given the varying genetic architecture of the quantitative traits we assessed here, we compared the PRS accuracy we observed with the accuracy that would be predicted from theoretical models.^51^^,^^52^ We calculated the predicted PRS accuracy for the European ancestry individuals from the UK Biobank according to the Daetwyler equation below, where *E* is the predicted accuracy, *h*^*2*^ is the heritability estimates, *M* is the number of independent SNPs (i.e., total number of trait-associated SNPs from LD clumping with p-value <1), and *N* is the sample size (∼350,000 individuals).

$$E\left(R^{2}\right)\approx \frac{h_{M}^{2}}{1+\frac{M}{Nh_{M}^{2}}}$$

#### Meta-analysis

We used plink2 to conduct inverse variance-weighted meta-analysis across GWAS summary statistics with the --meta-analysis option.

#### LD reference panels and clumping

All PRS calculations required an LD panel for clumping. Our analyses used in-sample LD where feasible and reference panel data as a proxy with ancestry matching from the 1000 Genomes Project phase 3 data when individual-level data were unavailable. We weighted the ancestral representation of each population per trait matching at the continental level. We matched individuals as follows.

**Table:** 

Cohort	1000 Genomes phase 3 reference data
BBJ	East Asian (EAS)
UK Biobank	European (EUR)
Uganda Genome Resource (UGR)	African (AFR)
PAGE	Proportional weighting of AFR, EAS, AMR (depending on trait, see Table S6 description for more detail)

We then used the maximal number of individuals available when weighting proportionally to construct this reference panel. For example, in the meta-analysis of height across the UK Biobank, Biobank Japan (BBJ), and Population Architecture Using Genomics and Epidemiology (PAGE) cohorts, UK Biobank has the largest sample size in the discovery cohort (n = 350,353), so all Europeans from 1000 Genomes were included in the reference panel (n = 503), then a random sampling of EAS, AFR, and AMR individuals were included proportionally to the overall diversity of the discovery cohorts in the meta-analysis.

## Results

Our study uses both simulation-based and empirical approaches to evaluate the generalizability of PRS across diverse African ancestry populations. Abbreviations are in Table S1, and a summary of datasets used in this study is shown in Table S2.

### Simulated generalizability within and across diverse African populations

**Figure 2:**
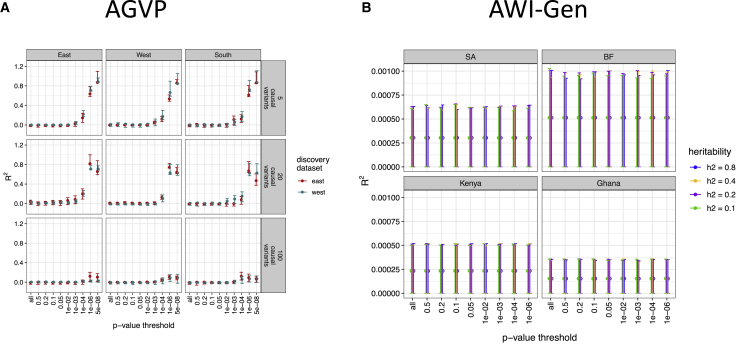
Simulated GWAS and polygenic scores indicate differential prediction accuracy across diverse regions of Africa (A) Predictive accuracy of the simulated quantitative trait in AGVP at the heritability of 0.8. The predictive accuracy was calculated for six categories of causal variants for the West and East discovery cohorts, across 10 p*-*value thresholds. Only the top three categories are shown here, the rest can be found in Figures S1–S4. (B) Predictive accuracy of simulated quantitative traits in AWI-Gen for various trait heritability rates across 10 p*-*value thresholds. The error bars represent the lower and upper limits of 95% confidence interval.

We used two separate simulation strategies for AGVP and AWI-Gen depending on their sample sizes (Figure 1). Given the limited sample size of the AGVP dataset, we opted to use the strategy previously used by Scutari et al.^11^ We simulated several quantitative traits with varying numbers of causal variants (n = 5; 20; 100; 2,000; 10,000; and 50,000) and heritability rates (h^2^ = 0.1, 0.2, 0.4, and 0.8), then conducted independent GWASs for each scenario in East and West African ancestry populations (materials and methods, Figures S1–S4). We calculated the prediction accuracy for PRSs derived from the GWAS summary statistics considering 10 different p-value thresholds within and across independent target populations from East, West, and South Africa. In general, ancestry-matched results with the sparsest and most heritable genetic architectures produced the highest prediction accuracy. As expected, prediction accuracy was highest with trait h^2^ = 0.8 and fewer than 100 causal variants (Figure 2A), as indicated by the highest *R*^2^ and the identification of genome-wide significant associations. Conversely, when the number of causal variants exceeded 100, prediction accuracy was negligible (Figure S4), as evidenced by no variants meeting the genome-wide significance threshold (i.e., p < 5e08). Prediction accuracy was highest with 5 and 20 causal variants (Figure 2A). The within-ancestry prediction at p-value threshold < 5e-08 and five causal variants were as follows: *R*^2^ = 0.86, p = 1.74 × 10^−74^ for East discovery - East target scores; *R*^2^ = 0.85, p *=* 9.9e-74 for West discovery - West target scores. We observed lower prediction accuracy with ancestry-mismatched discovery versus target cohorts at five causal variants and p-value threshold = 1e-6 (*R*^2^ = 0.66, p = 1.79e-42 for West discovery - West target scores, compared with *R*^2^ = 0.53, p = 1.29e-74 for East discovery - West target scores). The scores in the South target sample were comparable when using East- or West-derived summary statistics (*R*^2^ = 0.86, p = 5.19e-84 for West-derived summary statistics, and *R*^2^ = 0.86, p = 1.35e-83 for East-derived summary statistics).

For AWI-Gen, we used the commonly used infinitesimal simulation strategy for quantitative traits. We simulated quantitative traits by assigning genetic variant effects based on their minor allele frequency in accordance with the relationship between effects and minor allele frequency established by Schoech et al.^45^ We varied the trait heritability rates similar to the analysis done with AGVP and conducted GWASs for each trait (materials and methods, Figure 1). We calculated the prediction accuracy for PRS derived from the GWAS summary statistics considering 10 different p-value thresholds, as before, across independent target populations from Burkina Faso, Ghana, Kenya, and South Africa. Across heritability rates and target datasets, the PRS prediction accuracy was low and had confidence intervals that included zero (Figure 2B). The lack of PRS transferability in AGVP and AWI-Gen for traits with a polygenic architecture using two independent simulation strategies highlights that large-scale genetic studies in African populations are required to accurately predict phenotypes using genetic data and facilitate a better understanding of how PRS might transfer across African populations given the genetic diversity on the continent.

### PRS accuracies in South African populations

While our simulations have shown that PRSs generalize poorly across Africa due to substantial genetic diversity and differences across the continent, there is also considerable genetic and environmental diversity within regions and countries. We quantified PRS accuracy for a range of measured phenotypes in mothers genotyped in the DCHS cohort in South Africa, including several sociodemographic, physical/biomedical, and psychosocial risk traits (Table S3). The DCHS cohort consists of participants with multiple ancestry groups that include an admixed population with ancestry from multiple continents as well as a population with almost exclusively African population. These ancestry groups correlate with self-reported “Mixed” and “Black/African” ethnicities, respectively (Figure S5). We computed PRS for maternal height, depression, psychological distress, alcohol consumption, and smoking in DCHS overall, by ethnic group, and by ancestry within the Mixed ethnic group (materials and methods).

Across all genetically predicted phenotypes, only height was significantly predicted (Figure S6). We predicted height more accurately in the Mixed versus Black/African ethnic groups (*R*^2^ = 0.099, 95% bootstrapped CI = 0.012–0.18, p = 1.5e-7 versus *R*^2^ = 0.021, 95% CI = −0.031 to 0.043, p = 5.27e-3, respectively). We also expect that PRS accuracy increases with decreasing African ancestry within the Mixed ethnic group as has been shown previously in admixed African populations^53^; we find suggestive evidence consistent with this trend when partitioning the Mixed group into two bins along PC1 (*R*^2^ = 0.091, 95% CI = −0.04 to 0.17, p = 6.4e-4 in lower half of PC1 with more African ancestry versus *R*^2^ = 0.12, 95% CI = −9.0e-4 to 0.21, p = 5.7e-5 with more out-of-Africa ancestry), although small sample sizes limit definitive comparisons (n = 137 in each PC1 bin). Our results are consistent with variable prediction accuracy among diverse African ancestry groups within South Africa and insignificant prediction in African populations for all but the most heritable and accurately predicted traits elsewhere. Notwithstanding these findings, the sample used for these analyses is relatively small and does not represent the larger South African population and some of the traits are greatly impacted by pregnancy, for example, pregnant women are less likely to drink and smoke than the general public. In addition, in contrast to the discovery datasets that include males and females, DCHS is a female-only cohort that has both a lower and narrower age range, which could impact PRS accuracy for the traits where age plays a key role.

### Variable phenotypic and genetic similarities across the Uganda GPC and UK Biobank

#### Lower phenotypic correlations in the Uganda GPC suggest higher contributing environmental effects

We next investigated phenotypic similarities within and across the Uganda GPC and UK Biobank participants because these are two of the largest cohorts with dozens of traits measured in African ancestry individuals. We first considered overall cohort differences between these cohorts: the Uganda GPC enrolled participants using a house-to-house study design and generated genetic data on 5,000 adults from rural villages in southwestern Uganda,^39^ while the UK Biobank enrolled 500,000 people aged between 40 and 69 years in 2006–2010 from across the country (materials and methods^41^). Previous studies have reported higher rates of infectious diseases (e.g., HIV, hepatitis B and C) in the Uganda GPC than would be expected in the UK Biobank.^39^ There are many additional potential environmental explanations for mean shifts in phenotypes, such as dietary, food security, and age differences contributing to considerable BMI differences across cohorts (μ = 21.3 and σ = 3.8 in Uganda GPC versus μ = 27.4 and σ = 4.8 in the UK Biobank, p < 2.2e-16). To quantify comparisons while controlling for demographic differences for each of the 34 quantitative traits measured in both cohorts, we first mean centered each phenotype and regressed out the effects of age and sex within each cohort. Next, we then compared the distributions and variances of each phenotype across cohorts via Kolmogorov-Smirnov and F-tests, respectively (Table S4). Given the large sample sizes, all K-S tests were significantly different, with several phenotypes showing distributional and variance differences of considerable magnitude (Figure S7 and Table S4, e.g., Bilirubin, BASO, HbA1c, ALP, EOS, TG, and NEU).

**Figure 3:**
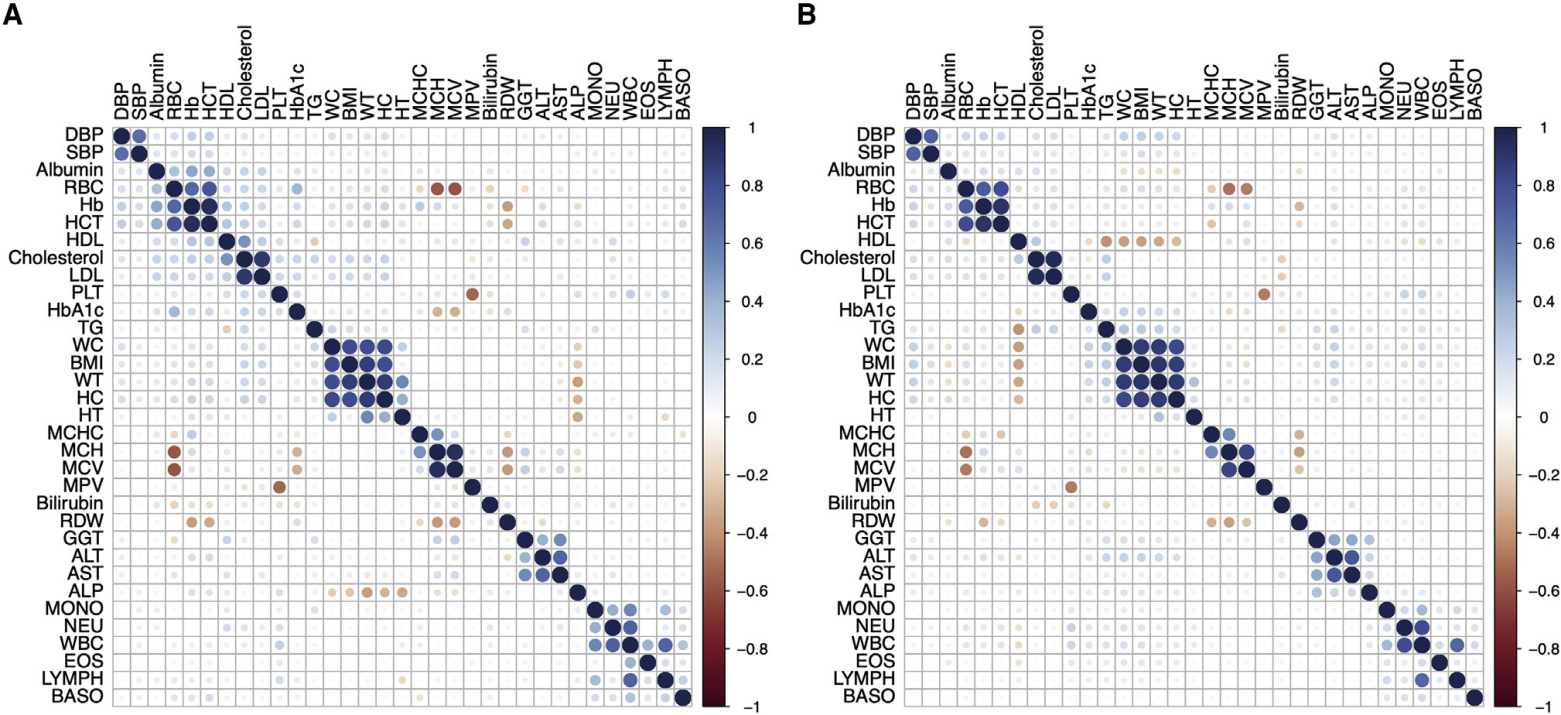
Phenotype correlations among 33 quantitative traits measured in the Uganda GPC data and the UK Biobank (A) Phenotypic correlations measured in traits in the Uganda GPC among unrelated individuals. (B) Phenotypic correlations in the unrelated UK Biobank European ancestry individuals. (A and B) Phenotypes were mean centered and adjusted for age and sex within each cohort prior to correlation analysis. The order of each phenotype correlation is determined by hierarchical clustering in the Uganda GPC.

We next analyzed how similar the relationships are between phenotypes across datasets. Similar trends emerge overall, with distances across variance-covariance matrices for these cohorts showing evidence of significant correlation (Mantel test *Z* statistic = 0.73, p < 1e-4). The correlations among phenotypes are slightly higher overall in the Uganda GPC than in UK Biobank, both among related and unrelated individuals (Figures 3B and S8). These findings are expected because of shared genetics and/or shared household environments contributing to more similar phenotypes.^54^ More specifically, we see consistent correlations among combinations of phenotypes including SBP and DBP; RBC, Hb, and HCT; Cholesterol and LDL; WC, BMI, WT, and HC; MCHC, MCH, and MCV; GGT, ALT, AST, and ALP; and MONO, NEU, and WBC with high overall correlations across these datasets for these traits (Figures 3A and 3B, see abbreviations in Table S1). Some pairs of traits, however, have significantly different correlations across datasets. The largest difference in phenotypic correlations across datasets is between ALP and WT (ρ = 0.11, p < 2.2e-16 in UK Biobank versus ρ = −0.36, p < 2.2e-16 in Uganda GPC).

Our next goal was to compare trait heritability estimates in the UK Biobank versus Uganda GPC data (materials and methods); however, the sample size and study design differences between these cohorts limited comparability using standard scalable approaches. Specifically, the household design of Uganda GPC included smaller sample sizes with more relatives in which family-based heritability estimates are most appropriate, whereas the large sample size and volunteer design in the UK Biobank makes SNP-based heritability estimates from unrelated individuals more appropriate. Figure S9 compares heritability estimates across traits in the UK Biobank versus Uganda GPC using these disparate approaches.^46^ As expected from the differences in the methods, study designs, and sample sizes, we find higher but noisier estimates in Uganda GPC for most traits, consistent with expectation from family-based versus unrelated heritability estimates across these two studies. While all of these factors fundamentally limit comparability of heritability estimates across these cohorts, we have also estimated heritability in both cohorts in unrelated individuals with consistent methodology using multi-component Haseman-Elston regression implemented in RHE-mc to improve comparability.^48^ These results showed higher heritability estimates in the Uganda GPC dataset that were not significantly correlated with heritability estimates from any ancestry group in the Pan-UK Biobank Project, consistent with a wide range of differences influencing these phenotypes across cohorts (Table S7, Figure S10). With these heritability estimates, we also estimated observed versus predicted PRS accuracy and find that predicted *R*^2^ tends to be higher than observed *R*^2^ (Figure S11).

#### African genetic risk predictions from European ancestry GWAS data are remarkably inaccurate

**Figure 4:**
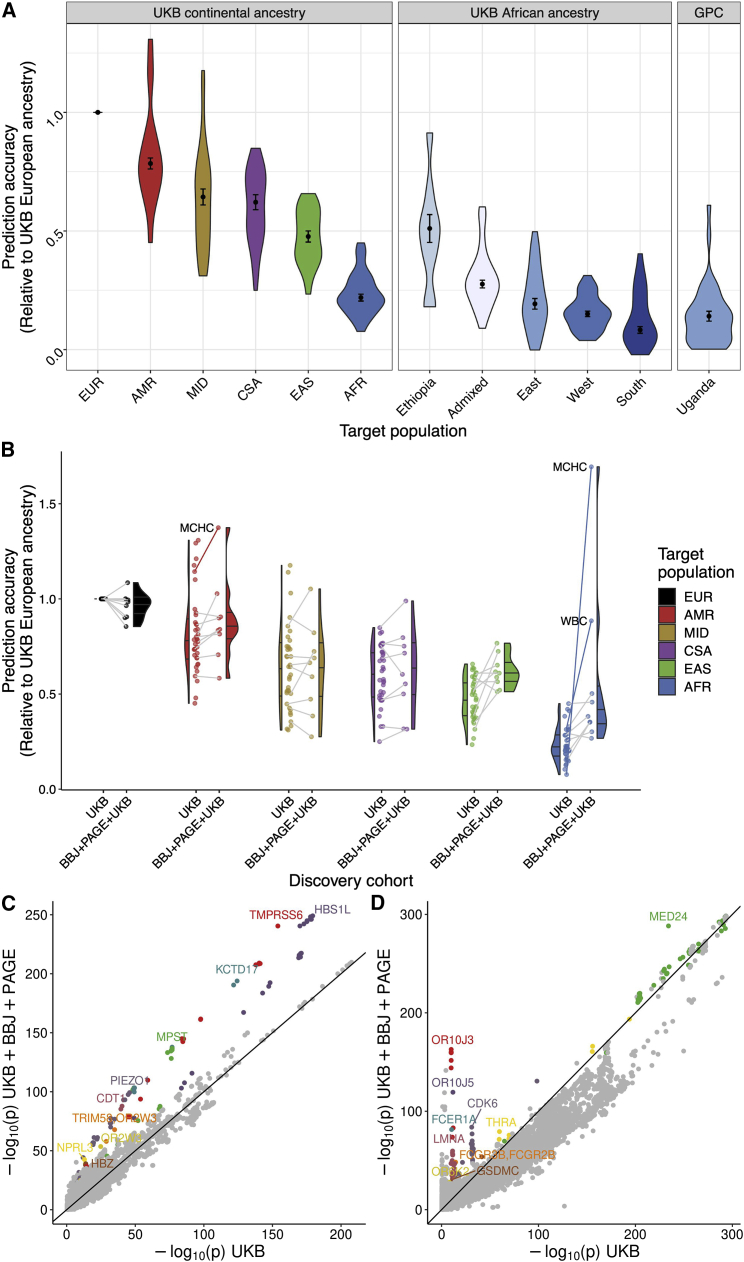
PRS accuracy and corresponding genetic variant contributions for up to 34 traits within and across diverse ancestries (A) PRS accuracy relative to European ancestry individuals in diverse target ancestries. Discovery data consisted of GWAS summary statistics from UK Biobank (UKB) European ancestry data. Target data consisted of globally diverse continental ancestries (including withheld European target individuals) and regional African ancestry participants from UKB, or unrelated individuals from the Uganda GPC cohort. Traits were filtered to those with a 95% confidence interval range in PRS accuracy <0.08. (B) PRS accuracy from a homogeneous versus multi-ancestry discovery dataset. GWAS discovery data consisted of summary statistics from UKB European ancestry data only or from the meta-analysis of UKB, BioBank Japan (BBJ), and Population Architecture using Genomics and Epidemiology (PAGE). Target populations are from the UKB. Lines connect the 10 traits available in both discovery cohorts to indicate how accuracy changed for the same trait in the UKB only versus meta-analyzed discovery data, while half violin plots show the distribution across all phenotypes in each discovery cohort. When lines are missing, the trait is absent in PAGE. Trait outliers are labeled in text and with solid lines. (A and B) Relative PRS accuracies are compared to the maximum for each trait in target samples withheld from discovery consisting of UKB European ancestry individuals. To simplify comparisons, only the polygenic scores with the highest prediction accuracy are shown here. Colors in these two panels correspond to the same continental ancestries. (C and D) Trait-specific genetic outlier plots. QQ-like plot showing p values in UKB only versus multi-cohort meta-analysis of UKB, BBJ, and PAGE. The 10 regions that are genome-wide significant in both dataset and show the most significant differences are colored and labeled for (C) MCHC, and (D) WBC.

To understand baseline trans-ancestry PRS accuracy using a typical approach, we predicted 32 traits in the Uganda GPC using GWAS summary statistics from the UK Biobank European ancestry individuals. While several traits were significantly predicted across ancestries, prediction accuracy was low for most traits (Figure S12); the most accurate PRS was for MPV (*R*^2^ = 0.036, 95% CI = 0.0069–0.063, p = 5.73e-7), while the average variance explained across all traits was less than 1% (mean *R*^2^ = 0.007). To assess the relative effects of ancestry versus cohort differences on decreases in prediction accuracy across populations, we next withheld 10,000 European ancestry individuals from UK Biobank for use as a target cohort, reran all GWASs, then used individuals with diverse continental ancestries in the UK Biobank as target populations (EUR = Europeans withheld from the GWAS, AMR = admixed American, MID = Middle Eastern, CSA = Central/South Asian, EAS = East Asian, and AFR = African, Figure S13), subcontinental African ancestries in the UK Biobank (Ethiopian, Admixed, South, East, West African ancestries, Figure S14), as well as the Uganda GPC (Figure 4A, Table S5).

Among continental ancestries, we computed *R*^2^ and 95% CIs for each trait (Figure S15), then computed median RA compared with Europeans and MAD across all traits (materials and methods). We predict these traits most accurately in EUR (RA = 1, MAD = 0), followed by AMR (RA = 0.784, MAD = 0.023), MID (RA = 0.643, MAD = 0.034), CSA (RA = 0.621, MAD = 0.031), EAS (RA = 0.477, MAD = 0.024), and AFR (RA = 0.219, MAD = 0.014) (Figure 4A). Because different PRS methodologies can improve overall prediction accuracy for some traits, we also compared our results using pruning and thresholding with PRS-CS; as described previously, different PRS methods may perform better for some phenotypes than others, but do not generally improve the relative loss of accuracy^19^^,^^55^ (Figure S16). We next compared prediction accuracy within African ancestry populations. Because some PRS accuracy estimates were noisy due to small sample sizes in UK Biobank Africans (especially Ethiopian and South African ancestry individuals, Table S5), we restricted analyses to those traits predicted with a 95% CI <0.08. Among these traits, we predicted most accurately those with Ethiopian ancestry (RA = 0.511, MAD = 0.059), followed by recently admixed individuals with West African and European ancestry (RA = 0.276, MAD = 0.016), East African ancestry (RA = 0.193, MAD = 0.023), West African ancestry (RA = 0.150, MAD = 0.012), and South African ancestry (RA = 0.083, MAD = 0.014) (Figure 4A). These results track with genetic distance as measured by F_ST_ (Table S8) and population history; the highest prediction accuracy identified in Ethiopians is expected given closer genetic proximity to European populations relative to other Africans due to back-to-Africa migrations influencing population structure there.^17^^,^^56^^,^^57^ The lowest prediction accuracy is in populations with southern African ancestry, consistent also with higher genetic divergence from European populations and more genetic diversity overall.^16^^,^^18^^,^^58^

Next, we quantified the proportion of loss of prediction accuracy (LOA, calculated as (1 − RA) ∗ 100%) due to MAF and LD in the subcontinental African ancestry groups in the UK Biobank. As expected, LOA followed an inverse trend to prediction accuracy, i.e., LOA increased with genetic distance between the discovery and target cohort (Figure S17). LOA was lowest in the Ethiopian group (median LOA = 26.22) and highest in the West group (median LOA = 40.11).

#### Lower prediction accuracy across ancestries than across cohorts

To compare prediction accuracy among similar ancestry participants from different cohorts, we next computed PRSs for 34 traits using GWAS summary statistics from UK Biobank Europeans in two target populations: UK Biobank participants with East African ancestry versus Uganda GPC. As expected, prediction accuracy in these populations is very low across all traits in both cohorts and only slightly higher in the UK East African ancestry individuals than in the Uganda GPC individuals (mean *R*^2^ = 0.017, SD = 0.013 versus mean *R*^2^ = 0.012, SD = 0.010, respectively, Figure S18). Across traits, the differences in PRS accuracy across cohorts but within the same ancestry (Figure S18A) are much smaller than the differences across ancestries but within the UK Biobank (Figure 4A, left and middle panels), indicating that ancestry has a larger impact on genetic risk prediction than cross-cohort differences analyzed here. Smaller effects on genetic prediction accuracy differences across cohorts may be attributable to environmental differences, such as higher rates of malnutrition and infectious diseases previously reported in Uganda and in the GPC.^39^^,^^59^

#### Improved African genetic risk prediction accuracy with multi-ethnic GWAS summary statistics

We next maintained the target populations but varied the discovery cohort to determine how more diverse GWAS impacts PRS accuracy for these phenotypes in diverse populations. Specifically, we computed PRS accuracy in diverse target populations in the UK Biobank (Table S5) using one of two discovery cohorts: the UK Biobank European-only cohort versus diverse discovery cohorts combined via meta-analysis (Table S6). Meta-analyzed GWAS summary statistics come from several cohorts, including the UK Biobank, BBJ,^60^ PAGE Consortium,^61^ and Uganda Genome Resource (UGR).^46^ For each trait, discovery cohort, and target cohort combination, we normalized the PRS *R*^2^ values from the p-value threshold that explained the maximum phenotypic variance with respect to the prediction accuracy in the European target cohort using UK Biobank summary statistics only, then computed RAs as before.

We find that prediction accuracy improves the most across populations when using a discovery cohort consisting of GWAS summary statistics meta-analyzed across the UK Biobank, BBJ, and PAGE cohorts (Figure 4B). To determine whether the improvement in prediction accuracy was due to the increase in sample size or the diversification of the GWAS discovery, we compared prediction accuracy across three discovery cohorts: 100,000 EUR individuals from GWAS summary statistics acquired from Martin et al., 2019a^10^, 350,000 EUR individuals, and multi-ancestry GWAS comprising UK Biobank, BBJ, and PAGE (Figure S19). We observe that the increase in discovery sample size from 100,000 to 350,000 EUR improves prediction accuracy differentially across populations (Figure S19A). When comparing prediction accuracy across the three discovery cohorts, the results show that increasing the sample size improves prediction accuracy across all ancestries, but more so for the EUR population. The multi-ancestry discovery cohort seemed to improve prediction accuracy in the non-EUR populations more than the increase in sample size in general, with the largest improvement in prediction accuracy observed for BMI in AMR and EAS populations and MCHC and WBC for the AFR population (Figure S19B).

**Figure 5:**
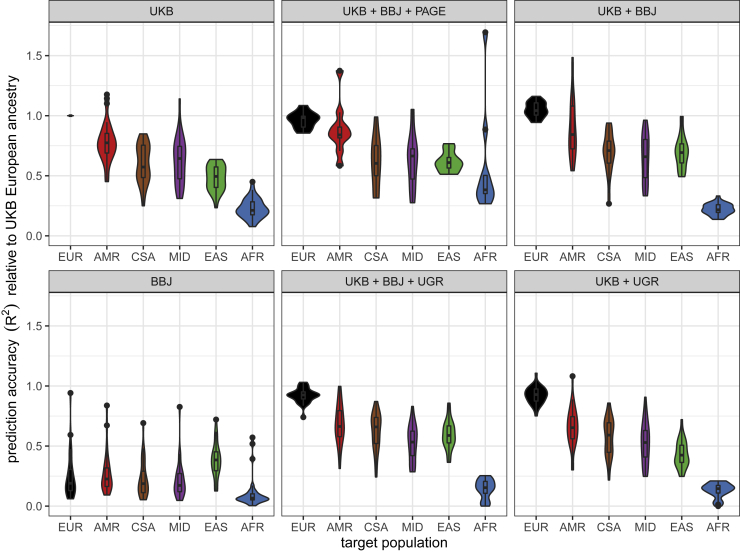
Relative PRS accuracy using the same target individuals and varying discovery cohorts All relative comparisons are with respect to accuracy in withheld EUR when predicting with UKB European GWAS summary statistics alone as the discovery cohort.

Surprisingly, meta-analyzing the UGR data with UK Biobank did not improve prediction accuracy for any population and most notably decreased accuracy in African ancestry target populations (discovery UK Biobank median RA = 0.22, UGR + UK Biobank median RA = 0.15, Figures 5 and S20). We hypothesize that (1) the relatively small sample size of UGR adds more noise than signal as indicated by the large error bars, and (2) the difference in effect sizes between UGR and UK Biobank, particularly for the less polygenic traits such as LDL (Figure S21) contributes to the noise. When predicting traits using the UK Biobank, BBJ, and PAGE meta-analysis as a discovery cohort, we find that prediction accuracy increases most for the AMR, EAS, and AFR target populations, which more closely resemble the ancestry patterns of PAGE and BBJ (Figure 4B). The meta-analysis conflates two factors that are known to improve prediction accuracy: increase in sample size and diversity in the discovery cohort. To determine which of these factors drove the gains in prediction accuracy in Figure 4B, we compared the prediction accuracy from 100K EUR individuals from UK Biobank, downsampled to match the size of BBJ, to that of the 350k EUR (for 17 overlapping phenotypes) and the multi-ancestry discovery (for five overlapping phenotypes). This comparison indicates that indeed increasing the discovery sample size generally improves prediction accuracy; however, it is the inclusion of diverse samples in the discovery cohort that improves prediction accuracy, especially for the populations represented in that cohort (Figure S19). These findings are consistent with ancestry-matched discovery data disproportionately improving prediction accuracy in the corresponding target population.^4^^,^^8^^,^^31^

#### Large-effect population-enriched genetic variants drive heterogeneity in polygenic score accuracy for blood panel traits

We find that PRS accuracy improvements from higher diversity in the discovery cohorts vary across traits, with the largest increases seen in MCHC and WBC particularly in AMR and AFR populations (Figure 4B). We searched for specific genetic loci that could explain this pattern by comparing the significance of genetic associations in UK Biobank alone versus the meta-analysis of UK Biobank, BBJ, and PAGE (Table S6). For MCHC and WBC in particular, the genetic variants contributing to these improved PRSs consist of several well-known population-enriched variants (Figures 4C and 4D). For example, genetic variants that disproportionately explain population-specific risk for MCHC include variants previously associated with hemoglobin concentration, including rs9399137 upstream of *HBS1L* and *MYB* in a study of sickle cell anemia (p = 5.24e-249 and β = 0.0783 in the meta-analysis),^62^ rs855791 in *TMPRSS6* (p = 3.49e-241, β = 0.0692),^63^^,^^64^ and rs551118 upstream of *PIEZO1* and *CDT1* (p = 5.18e-100, β = −0.0451)^65^ (Table S9). Associations with WBC tend to show more population-enriched associations as shown in the meta-analysis (Figure 4D), including rs3936197 in *MED24* (p = 5.18e-289, β = −0.0772), rs58650325 near the high affinity immunoglobulin (Ig)E receptor *FCER1A* that initiates the allergic response (1.57e-163, β = −0.097, also close to *OR10J3*), and rs11533993 in *CDK6* (p = 1.55e-84, β = −0.0799). Thus, genetic architecture and population genetic considerations are important to bear in mind when considering the generalizability of polygenic scores.

## Discussion

PRSs have been proposed as genetic biomarkers for use in preventive medicine,^66^^,^^67^ but are currently limited by low accuracy across populations especially in African ancestry populations.^4^^,^^6^ Through simulations and empirical work, this study has enabled unique insights into PRS transferability within and among diverse continental African populations as well as among African ancestry populations living in considerably different environments. Simulations will continue to play a crucial role in understanding and mitigating biases, but the small sample size of existing genetic studies in African populations have limited the simulation designs that are even possible with realistic population structure across the African continent in this study. The AGVP dataset used for the first set of simulations was too small to use a typical infinitesimal simulation strategy. As a result, we simulated phenotypes with variants with large effects—a scenario that is inconsistent with the genetic architecture of most polygenic traits. While the simulation done with the AWI-Gen dataset represents a scenario that is more realistic for complex traits, the findings re-emphasize that the paucity of large genetic samples in non-European ancestry populations limits simulation designs. Future studies could simulate new individuals from observed allele frequencies or from larger scale genetic datasets as they are made available. Despite these limitations, the simulation work done here provides a framework for simulation designs within the current sample size confines and what can be expected from these simulations in African populations.

We demonstrate looming challenges for applying current PRS in African ancestry populations—because relatively few genetic studies have been conducted in African populations coupled with the lack of out-of-Africa population bottlenecks, PRS accuracy is low but widely variable. Differences in PRS accuracy across diverse African ancestries from different regions can be larger than across out-of-Africa continents. This is particularly problematic, as widely used algorithms that guide health decisions already have ingrained racial biases,^68^ warning of compounding challenges with implementation. We demonstrate that there are clear steps the field can take to work against these biases. Specifically, including ancestrally diverse populations in GWASs at considerably larger sample sizes, discovery cohorts improve accuracy for all populations and especially underrepresented populations more than conducting similarly sized studies with only European ancestry cohorts.

Another advantage of using GWASs from globally diverse populations to compute PRS is the routine inclusion of population-enriched variants. Clear examples such as African-enriched variants in *APOL1* and *G6PD* have been shown to contribute to especially high risk of chronic kidney disease and to missed diabetes diagnosis, respectively.^69^^,^^70^ These examples highlight the importance of studying diverse populations to predict genetic risk of disease equitably by aggregating variants across the spectrum of allele frequencies and effect sizes in different populations. Relevant to the traits studied in genetic analyses here, hematological differences such as anemia are more common in lower income countries in Africa and in African ancestry populations elsewhere compared with European ancestry populations in high-income countries, particularly among older individuals. These hematological differences potentially arise in part due to genetic variation as well as the higher prevalence of infectious diseases and pathogens, poorer nutritional status, and altitude.^71^^,^^72^ Here, we show that variants influencing risk of beta thalassemia disproportionately increase PRS accuracy for hemoglobin variation particularly in African ancestry populations. The inclusion of population-enriched variants in PRS could eliminate genetic justifications for race-based medicine, which problematically reinforces implicit racial biases by overemphasizing the link between genetics and race despite the fact that there is more genetic variation within than between ancestral populations.^73^ However, for this to be possible, genetic data would have to be available for all populations at scale—an ideal that is still a ways off.

In addition to reduced PRS accuracy with ancestral distance from GWAS cohorts, genetic nurture, social genetic, and environmental effects can also contribute to low portability of PRS across populations,^23^^,^^74^ with some interventions modulating health along PRS strata.^75^ In this study, however, ancestry appears to have a larger effect on portability than cohort differences overall. An important distinction when comparing the magnitude of these and other non-genetic effects in other studies is that the traits most accurately genetically predicted here were primarily anthropometric and blood panel traits. When analyzing traits with more sociodemographic influences in increasingly diverse populations, population stratification, confounding, and study design considerations are thornier issues.^22^^,^^76^^,^^77^ PRS accuracy comparisons across ancestrally similar but environmentally diverse populations are especially important for medically actionable traits. For example, particularly low PRS portability for triglycerides (TG) from European to the Uganda GPC resulted at least in part from effect size heterogeneity that has previously been connected to pleiotropic and gene ∗ environment effects; specifically, most non-transferable genome-wide significant associations with TG showed pleiotropic associations with BMI in European but not Ugandan individuals.^78^

While PRSs currently have limited portability, increased diversity in genetic studies is already decreasing prediction accuracy gaps across populations.^31^^,^^78^^,^^79^ This is consistent with causal genetic effects tending to be similar across populations but with LD and allele frequency differences modifying marginal effect size estimates.^4^^,^^7^^,^^8^ This is also consistent with trans-ethnic genetic correlations tending to be close to or not significantly different from 1.^80^^,^^81^ The most rapid path to closing gaps in PRS transferability is to increase the inclusion of GWAS participants from populations most divergent from those already routinely studied. As empirically demonstrated here, when comparing PRS accuracy calculated from diverse cohort meta-analysis versus data from Europeans only, large-scale GWASs with diverse African populations will rapidly reduce portability gaps across global populations because they have the most genetic diversity, most rapid linkage disequilibrium decay, and highest genetic divergence from the best studied populations. Major efforts under way, such as the Human Hereditary and Health in Africa Initiative, PAGE, All of Us, and NeuroGAP programs,^61^^,^^82^^,^^83^^,^^84^^,^^85^ are especially promising for rectifying current PRS gaps and missed scientific opportunities by increasing inclusion of diverse African participants.

Beyond expanding on diversity by increasing the number of study participants in large-scale studies, it is equally important to diversify researchers working on genomics studies. Currently, the vast majority of researchers in genomics studies are of European ancestry,^86^^,^^87^^,^^88^ paralleling the over-representation of European ancestry individuals in genomic studies. The exclusion of African researchers leads to the disparity in research leadership and reduced scientific output from African researchers.^89^ Efforts such as the Global Initiative for Neuropsychiatric Genetics Education and Research (GINGER) program,^90^ which provides mentorship and training for early-career investigators on the African continent (particularly in Uganda, Kenya, Ethiopia, and South Africa, including several of this study’s authors), are important in moving toward a more inclusive and representative research community.

### Conclusion

Previous studies that have examined PRS accuracy across globally diverse ancestry groups have demonstrated that accuracy is lowest in African ancestry samples. However, the extent to which this accuracy varies within African ancestry populations has not been previously investigated. Our findings that prediction accuracy varies by African ancestry populations is a clear reflection of the vast genetic diversity of the continent. It is therefore critically important to create well-powered GWASs that reflect the full range of diversity within Africa.

## Data and code availability

All data used in this study are publicly available. Data from the African Genome Variation Project was accessed by combining EGAD00010001045, EGAD00010001046, EGAD00010001049, EGAD00010001050, EGAD00010001051, EGAD00010001052, EGAD00010001053, EGAD00010001054, EGAD00010001055, EGAD00010001056, EGAD00010001057, and EGAD00010001058. The Drakenstein Child Health Study is committed to the principle of data sharing. De-identified data will be made available to requesting researchers as appropriate. Requests for collaborations to undertake data analysis are welcome. Uganda GPC genetic data used in this paper were accessed through EGAD00010000965 and phenotype data were accessed via sftp from EGA (reference: DD_PK_050716 gwas_phenotypes_28Oct14.txt). We accessed data from the UK Biobank with application 31,063. BioBank Japan summary statistics were accessed from http://jenger.riken.jp/en/result. GWAS summary statistics for the Population Architecture using Genomics and Epidemiology (PAGE) study were accessed through the NHGRI-EBI GWAS Catalog (https://www.ebi.ac.uk/gwas/downloads/summary-statistics).

All code used in analysis is available here: https://github.com/armartin/africa_prs.

## Web resources

European Genomic-Phenome Archive, https://ega-archive.org.

Drakenstein Child Health Study, http://www.paediatrics.uct.ac.za/scah/dchs.

BioBank Japan summary statistics, http://jenger.riken.jp/en/result.

Population Architecture using Genomics and Epidemiology (PAGE), https://www.ebi.ac.uk/gwas/downloads/summary-statistics.

## Supplemental information

Data S1. Tables S1–S9

Document S2. Article plus supplemental information
